# Complication rates in real-time ultrasound-guided vs static echocardiography-guided pericardiocentesis: a cohort study

**DOI:** 10.1186/s44156-025-00071-6

**Published:** 2025-04-01

**Authors:** Virginia Zarama, Carlos E. Vesga, John Balanta-Silva, Mario M. Barbosa, Jaime A. Quintero, Ana Clarete, Paula A. Vesga-Reyes, Juan Carlos Silva Godinez

**Affiliations:** 1 Departamento de Medicina Crítica, Fundación Valle del Lili, Cali, Colombia; 2 Facultad de Ciencias de la Salud, Universidad Icesi, Cali, Colombia; 3 Departamento de Cardiología, Fundación Valle del Lili, Cali, Colombia; 4 Centro de Investigaciones Clínicas (CIC), Fundación Valle del Lili, Cali, Colombia; 5 Harvard T.H. Chan School of Public Health, PPCR Program, Boston, USA; 6 Escuela Nacional Colegio de Ciencias y Humanidades, Universidad Nacional Autónoma de México, Ciudad de Mexico, México

**Keywords:** Ultrasound-guided, Real-time, Pericardiocentesis, In-plane, Echocardiography

## Abstract

**Background:**

Static echocardiography-guided pericardiocentesis, the current standard of care, uses a phased-array probe to locate the largest fluid pocket, marking the safest entry site and needle trajectory. Nevertheless, real-time needle visualization throughout the procedure would potentially increase success and decrease complications. The aim of this study was to assess the complication rates of the real-time in-plane ultrasound-guided technique compared to the traditional static echocardiography-guided pericardiocentesis.

**Methods:**

All adult patients who underwent pericardiocentesis in a tertiary care hospital from January 2011 to June 2024 were identified. The incidence of total complications of the real-time, in-plane, US-guided pericardiocentesis versus the static echocardiography-guided technique was compared using a regression model with overlap weighting, based on propensity scores, to adjust for confounding factors.

**Results:**

A total of 220 pericardiocentesis were identified, 91 with real-time, in-plane US-guided technique and 129 with a static echo-guided approach. The overall rate of total complications was 5.5%, with no significant difference between both techniques (IRR 1.06 [95% CI 0.98 to 1.16, p = 0.163]). Only one major complication was reported with the in-plane technique (pulmonary edema) compared to four major complications in the echo-assisted approach (three cardiac injuries and one injury to thoracic vessels), all of which required emergency surgery. The success rate was higher in the real-time in-plane US-guided procedures (97%) compared to the static echo-guided approach (93%).

**Conclusions:**

In this single-center retrospective cohort study, real-time in-plane, US-guided pericardiocentesis technique was safe, and the rate of total complications was not significantly different from a static echo-guided approach. The low rate of major complications and high success rate underscores the potential use of this technique in emergency situations by well-trained physicians. Future studies are warranted to thoroughly assess the potential benefits of the real-time approach.

**Supplementary Information:**

The online version contains supplementary material available at 10.1186/s44156-025-00071-6.

## Background

Pericardial effusion is a condition frequently encountered in emergency and critical care settings. The primary cause of pericardial effusions requiring drainage is malignancy, followed by cardiac surgery and complications from catheter-based procedures [1]. Other less common causes include kidney disease, autoimmune disorders, trauma, and infections [1–3]. However, pericardiocentesis carries risks, ranging from major complications such as cardiac laceration, injuries to thoracic or coronary vessels, pneumothorax, and even death, to minor complications including vasovagal reactions, catheter obstruction or pleuro-pericardial fistulas [4].

The pericardiocentesis technique has evolved from the initial surgical open approach to a percutaneous blind approach, which has been associated with significant morbidity and mortality rates of 60% and 5% respectively [5–8]. Subsequently, with the introduction of two-dimensional echocardiography, the static echocardiography-guided technique has become the most common approach and it is currently considered the standard of care [8–11]. First described at the Mayo Clinic in 1979, the static echo-guided technique uses a phased-array probe to locate the largest fluid pocket and identify the safest approach, marking the ideal entry site and needle trajectory [8, 12]. A large cohort study from the same institution, including 1127 therapeutic echo-assisted pericardiocentesis over 21 years, reported an overall complication rate of 4.7% (major, 1.2%; minor, 3.5%) [1].

With technological advancements, the widespread availability of portable ultrasound machines, and increased training in ultrasound-guided procedures, clinicians are increasingly adopting a real-time ultrasound guidance in pericardiocentesis. This approach provides real-time visualization of the needle tip throughout its trajectory, promoting a safer entry to the pericardial sac. The in-plane technique aligns the needle’s axis with the plane of the ultrasound beam, allowing continuous visualization of both the needle shaft and tip during insertion [13]. By continuously assessing the distance between the needle tip and the moving cardiac structures, this technique potentially enhances procedural safety.

In 2013, a parasternal real-time in-plane ultrasound technique for pericardiocentesis using a curvilinear probe was described in a case-report, [14]. In 2016, a prospective cohort was published using a bracket attached to the phased-array probe to guide needle insertion [15]. Subsequently, the introduction of a high-frequency linear probe to enhance needle visualization with a real-time, in-plane technique was reported in small children [16, 17]. Finally, in 2018, an in-plane, medial-to-lateral approach using a linear probe in a cohort of 11 adult patients presenting to the emergency department with cardiac tamponade was reported, achieving a 100% success rate with no complications [18].

The use of a real-time, in-plane ultrasound-guided pericardiocentesis technique with a high-frequency linear probe could further reduce the rate of major complications encountered with static echo-guided procedures. More importantly, it offers a safe and timely option for emergency and critical care physicians to perform pericardiocentesis in cardiac tamponade situations. Given their training in thoracic and focused cardiac ultrasound, as well as in real-time ultrasound-guided procedures, like central venous catheterization, these physicians are well-positioned to use this technique effectively [19–22].

At our institution, the real-time, in-plane ultrasound-guided pericardiocentesis technique has been progressively integrated over the past 6 years, and it is now widely embraced by the emergency, intensive care and cardiology departments. Given the increased adoption of this method, particularly in high-stakes scenarios such as cardiac tamponade, it is important to assess whether the real-time, in-plane approach offers advantages over the traditional static echo-guided pericardiocentesis in terms of reducing complication rates. Therefore, the aim of this study is to compare the complication rates of the real-time, in-plane ultrasound-guided technique to the traditional static echo-guided pericardiocentesis in a real-world clinical setting.

## Methods

We retrospectively identified all adult patients aged 18 years and older who underwent pericardiocentesis between January 2011 and June 2024, in Fundación Valle del Lili, a university tertiary-care hospital in Cali, Colombia. The study protocol was approved by the local Ethics and Biomedical Research Committee for studies involving human subjects. Given the retrospective nature of the study, the requirement for informed consent was waived. The Strengthening the Reporting of Observational Studies in Epidemiology (STROBE) checklist is included in the electronic supplement (Supplementary Material File 1).

The hospital’s electronic health record database was searched using the pericardiocentesis procedure code and the pericardiocentesis kit medical supply code to identify all patients who underwent the procedure. A standardized data extraction form was used for retrospective chart review. Clinical and laboratory records were carefully reviewed by three investigators (JQ, JB and PV) after standardizing the data extraction method. Complete information on demographics, baseline clinical characteristics, comorbidities, procedural details, operator characteristics, and complications was recorded.

### Pericardiocentesis techniques

#### Static echo-guided pericardiocentesis

A phased-array transducer is used to identify and mark the optimal entry site, ensuring that the largest pericardial fluid accumulation is closest to the surface while avoiding vital structures. It also determines the ideal direction and depth for the intended needle insertion, based on the angulation of the phase-array probe and the distance from the skin to the pericardial sac, as outlined by Tsang et al. [12]. Following this, the needle is inserted in a straight trajectory until pericardial fluid is aspirated. After inserting the needle and aspirating pericardial fluid, agitated saline is injected through the needle while performing simultaneous echocardiography to confirm the intrapericardial position. Then, a guidewire is advanced to the pericardial space, and the pericardial drain or pig-tail catheter is introduced through the wire using Seldinger’s technique [1, 10, 12]. Common access points include the apical or para-apical, parasternal, and subxiphoid regions.

#### Real-time, in-plane, US-guided pericardiocentesis

**Fig. 1:**
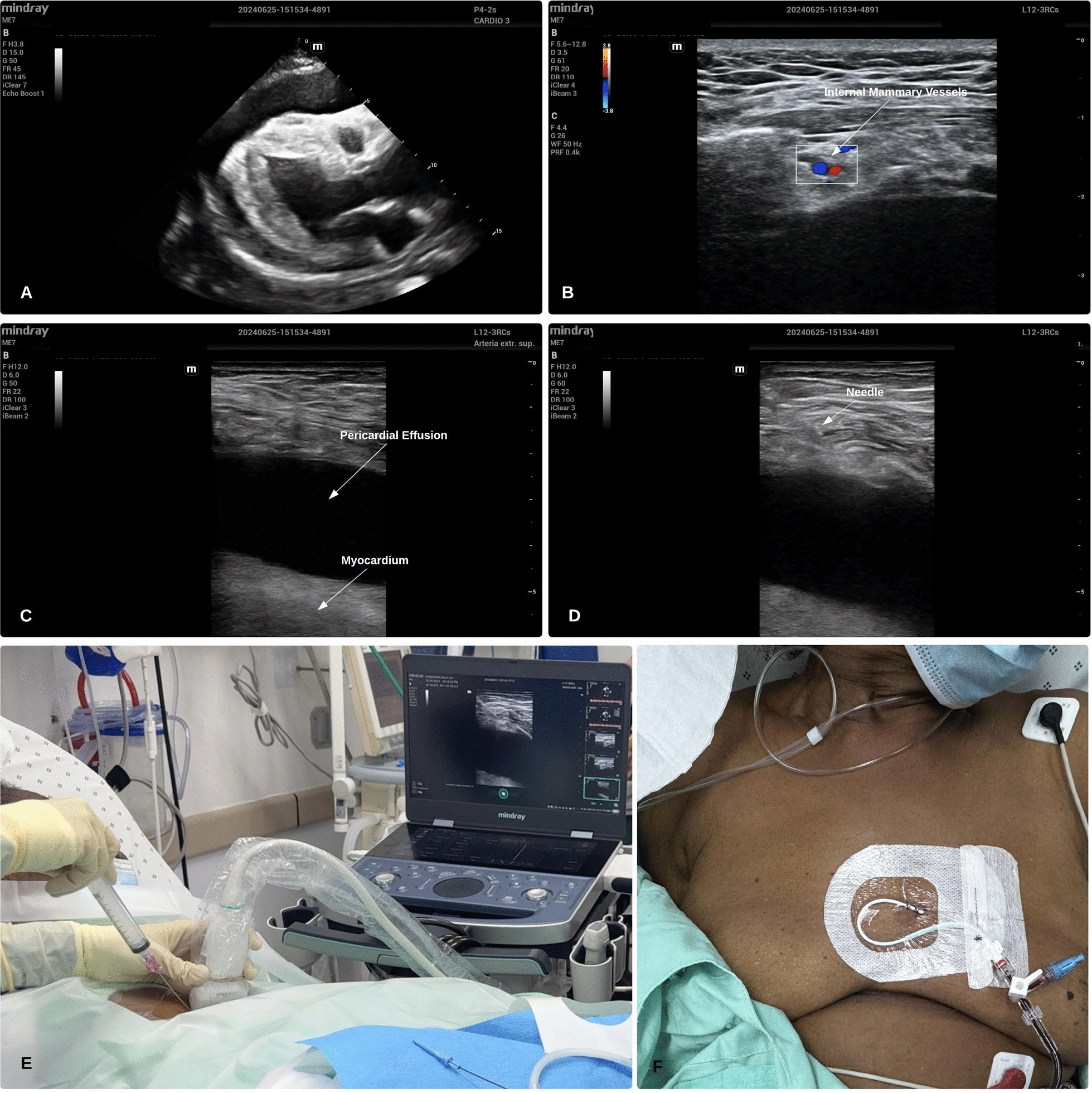
Real-time, in-plane US-guided pericardiocentesis. A phased-array probe is used to locate the largest pericardial fluid accumulation in the parasternal o para-apical windows **a**. A high frequency linear probe is used to locate the internal thoracic vessels **b**, to estimate the distance from the skin to the pericardial sac **c** and to guide needle trajectory using a real-time in-plane technique, until the needle tip reaches the pericardial sac **d**, **e**. Catheter secured to the skin with a standard suture technique **f**

A phased-array probe is initially used to identify the largest pericardial fluid accumulation and assess the depth of pericardial sac, typically in the parasternal or para-apical windows (Fig. 1a). Following this, a linear high frequency probe is employed to the estimate the distance from the skin to pericardial sac and to locate the safest entry point, avoiding internal thoracic vessels and selecting an area where the distance from the moving heart inside the pericardial sac is greatest. To locate the internal thoracic vessels, the linear transducer is positioned along the left parasternal line, parallel to the rib axis. The vessels can then be visualized in their short axis anterior to the pleural line or the pericardial space (Fig. 1b) [23]. After performing standard sterile skin preparation, a sterile cover is applied to the linear probe, ensuring it remains positioned parallel to the rib axis. An in-plane technique is used for real-time visualization of the needle trajectory as it advances medially to laterally toward the pericardial sac (Fig. 1c–e, Supplementary Material file 2, 3: Videos). This approach also facilitates verification of the guidewire within the pericardial space and confirmation of catheter placement using agitated saline.

### Outcomes

Our primary outcome was the incidence of total complications. Major complications were defined as events requiring surgical, endovascular, or other invasive interventions, or those associated with hemodynamic instability. Minor complications were categorized as those not requiring invasive intervention and were managed with monitoring or clinical observation. In line with previous studies, major complications included cardiac chamber perforation or laceration, pericardial/epicardial thrombus requiring surgery, ventricular arrythmias, pneumothorax necessitating thoracostomy, injury to intercostal or coronary vessels, pulmonary edema, or systemic infections. Minor complications included vasovagal reactions, non-sustained supraventricular arrythmias, pericardial catheter occlusion, pleuro-pericardial fistula or, small pneumothorax [1, 4, 5, 12, 24]. Pericardiocentesis was considered successful if the pericardial space was entered, fluid was drained, and tamponade relief was achieved.

### Statistical analysis

Continuous variables were expressed as medians and interquartile ranges (IQR) and categorical variables were expressed as frequencies and percentages. The Shapiro–Wilk test was performed to determine the normality of data distribution.

Given the observational nature of our study, we employed a propensity score to mitigate confounding. Propensity scores were estimated using a logistic regression model, where the treatment assignment was regressed on the sex and age of the patient, blood urea nitrogen, abnormal coagulation, thrombocytopenia (platelet count < 50,000), type of ward (general ward vs. emergency room vs. ICU), hemodynamic stability, initial drainage volume, and operator experience. These variables were chosen based on clinical relevance and prior literature. To account for the distribution of the propensity scores, we used overlap weights, which emphasize in individuals for whom there is substantial clinical equipoise regarding treatment assignment. This method also enhances the efficiency and precision of the estimated treatment effects by focusing on the population where the treatment effect is most identifiable [25–27]. Overlap weights are defined as the minimum of the propensity score (ps) and its complement (1—ps), ensuring a balanced and unbiased comparison between the treated and untreated groups [25]. The distributions of propensity scores for the control group, treated group, combined sample, and overlap weighted sample were visualized using kernel density plots (e-Fig. 1). The graph demonstrates the density of propensity scores by treatment status, illustrating how overlap weighting achieves better balance between the different technique groups. After obtaining the overlap weights, we analyzed the primary outcome of interest, which is the occurrence of complications, using a weighted Poisson regression model with robust standard errors.

Coagulation tests were available for 44% of the patients. To explore the potential influence of coagulation abnormalities on procedural outcomes, abnormal coagulation was defined as a prothrombin time or partial thromboplastin time exceeding twice the upper limit of normal, an INR ≥ 2.0, or the use of anticoagulant medications. For cases with missing coagulation data, normal coagulation status was assumed. A sensitivity analysis was conducted to assess the robustness of this assumption using multiple imputation with chained equations, as well as a worst-case scenario where all missing coagulation data were considered abnormal and complete case analysis. A detailed description of missing variables and justification for missing at random assumption is included in Supplementary Material file 1.

## Results

**Fig. 2:**
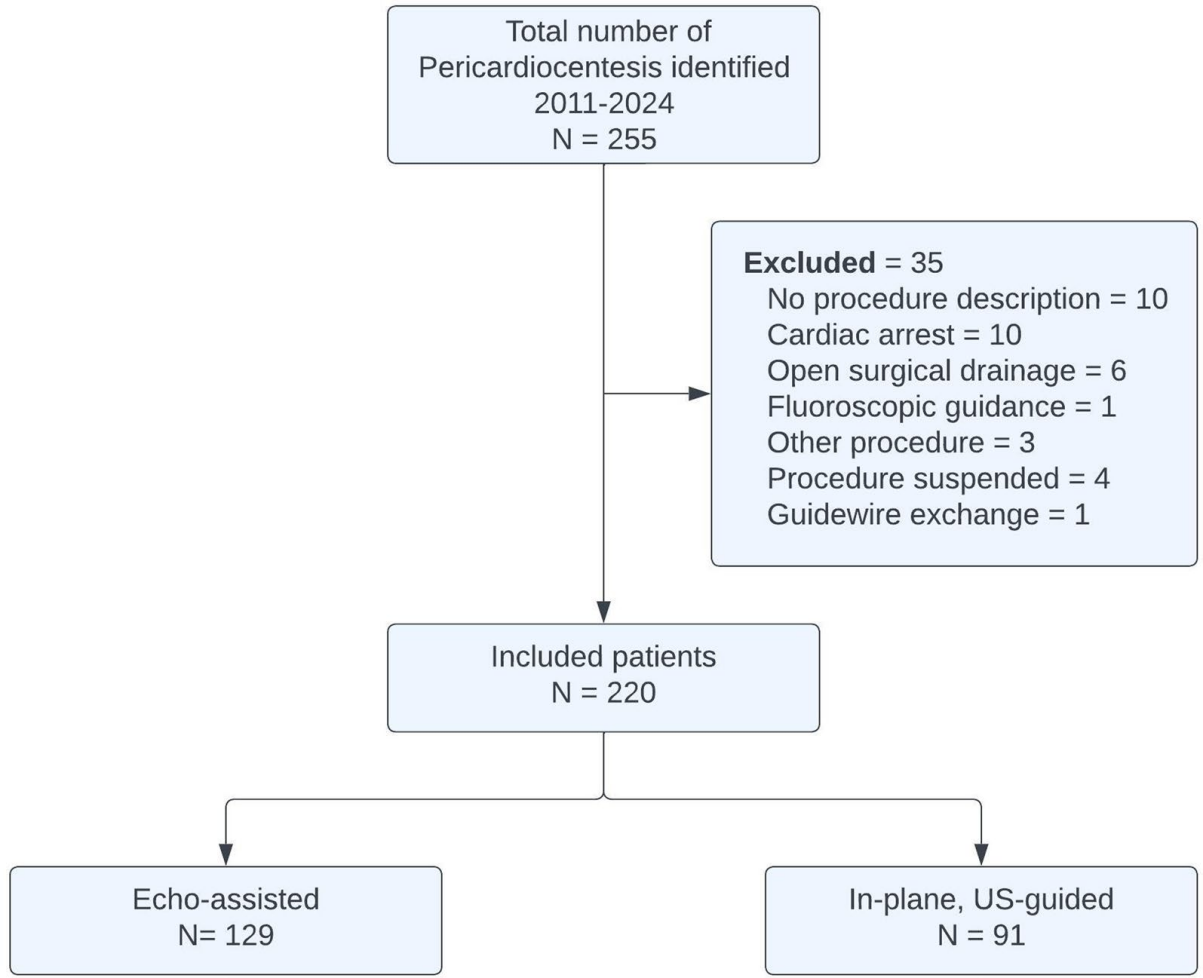
Flow diagram

From January 2011 to June 2024, 255 pericardiocentesis procedures were identified. Exclusion criteria are outlined in the flow diagram (Fig. 2). The analysis included 220 pericardiocentesis procedures performed on 209 patients. Among these, 91 were done with the real-time US-guided technique and 129 with the static echo-guided approach.

Demographics and general characteristics of the overall population are described in Table 1. Median age was 56 years, and a similar distribution of comorbidities was found between the two groups. Cancer was the primary etiology of pericardial effusions, followed by post-cardiac surgery or catheter-based procedures. Most pericardiocentesis took place in the ICU (58%), where the static echo-guided technique was favored (69%). Conversely, 36% of the procedures were performed in the emergency department (ED), primarily using the real-time US-guided technique (62%). Hemodynamic instability was more predominant in patients subjected to the static echo-guided pericardiocentesis (79%), compared to approximately half the patients in the in-plane US-guided groups.

**Table 1:**
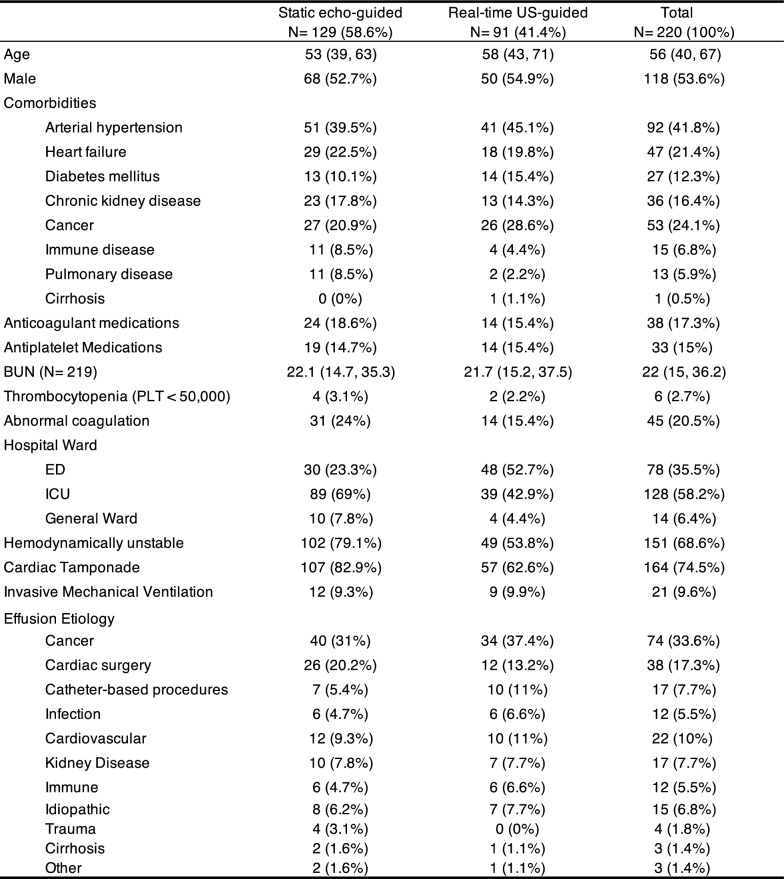
Baseline characteristics

Values are reported as median (Q1, Q3) or %

Echo echocardiography, US ultrasound, BUN blood urea nitrogen, PLT platelet count, ED emergency department, ICU intensive care unit

**Fig. 3:**
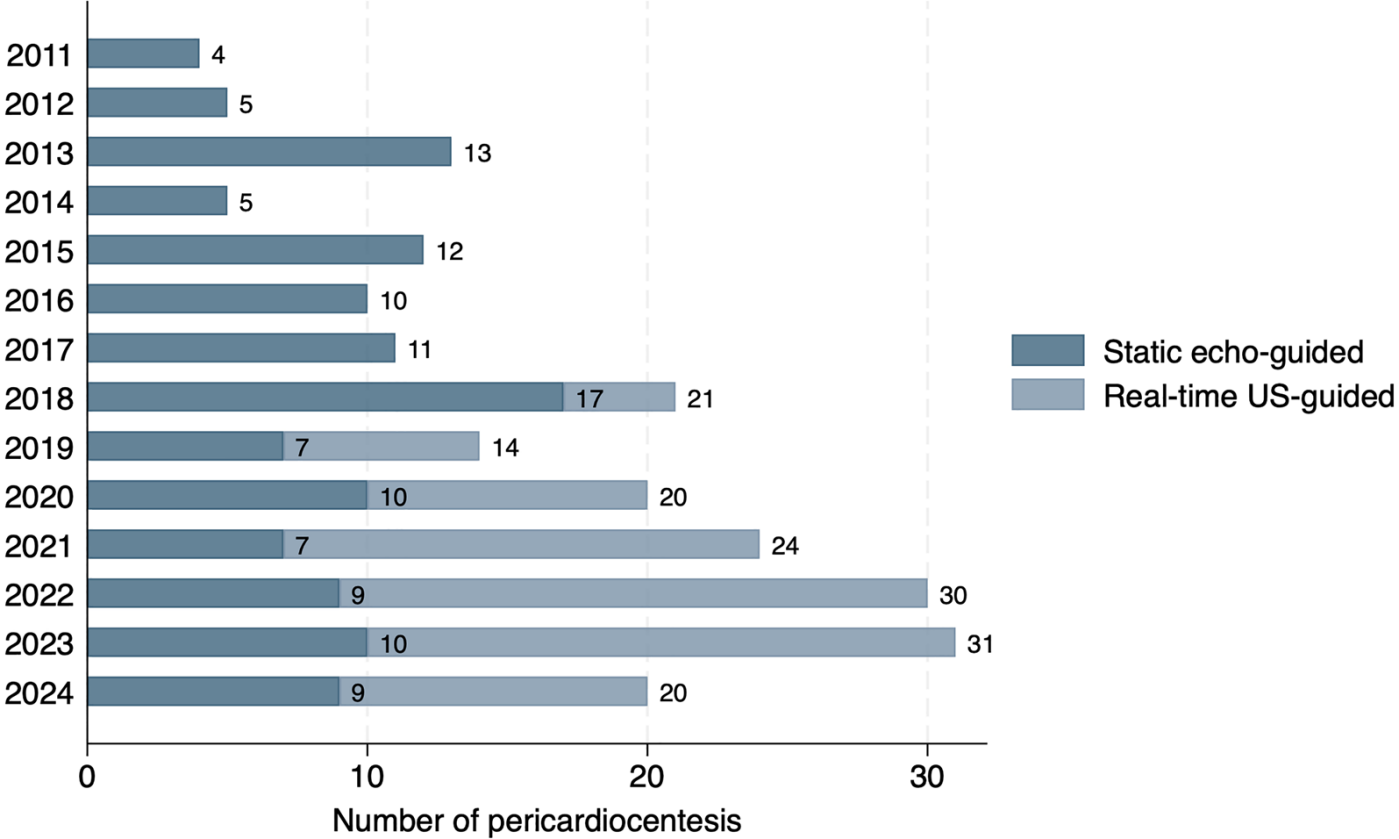
Temporal trend in the use of static echo-guided and real-time US-guided pericardiocentesis

Procedural characteristics are outlined in Table 2. Most procedures were performed by cardiologists (94%) with emergency physicians conducting the remaining 6%. The static echo-guided technique was primarily performed by cardiologists, while emergency physicians predominantly used the real-time US-guided approach. The apical/para-apical access site was the most common in the echo-guided technique (75%), whereas the left parasternal access site was predominant in the real-time US-guided procedures (97%). As expected, more than half (56%) of the real-time ultrasound-guided procedures were conducted by physicians with limited experience in the technique, defined as fewer than 10 or between 10 and 24 previous procedures. In contrast, 68% of the static echo-guided procedures were performed by more experienced physicians who had completed over 25 prior procedures. Over the years, there has been a growing trend in favor of the real-time US-guided technique compared to the static echo-guided approach (Fig. 3).

**Table 2:**
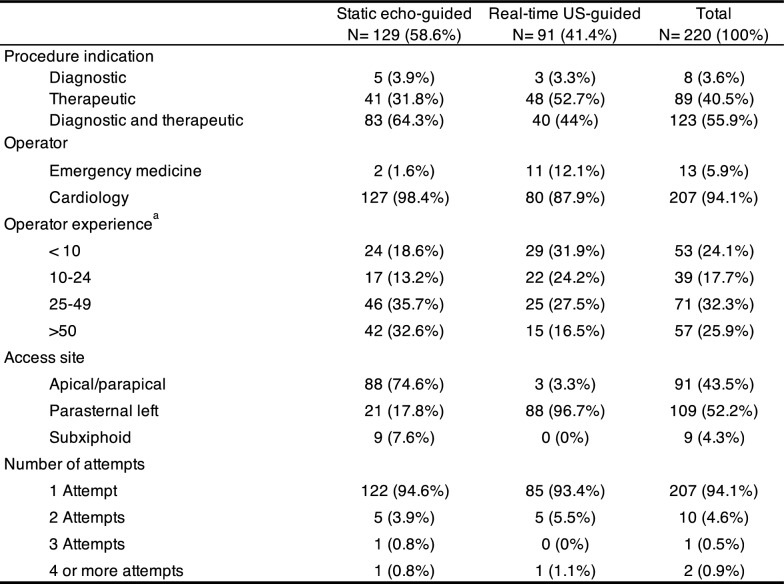
Procedural characteristics

PDS Pericardiotomy Decompression Syndrome

^a^Based on the number of previously performed pericardiocentesis with the specific technique

Procedural success was 93% in the static echo-guided procedures and 98% for the real-time, US-guided pericardiocentesis. The echo-guided group experienced eight complications (6.2%), four of which (3.1%) were classified as major complications (Table 3). These included three cardiac lacerations and one thoracic wall vessel injury, all requiring open surgical management. In the real-time US-guided group, there were four complications (4.4%), with one major complication (1.1%). This major complication involved a patient who developed pulmonary edema requiring tracheal intubation. The three (3.2%) minor complications included a small pneumothorax (< 10%) that did not require tube thoracostomy and was managed with observation, one catheter obstruction and a pleuro-pericardial fistula. Notably, there were no deaths in this cohort.

**Table 3:**
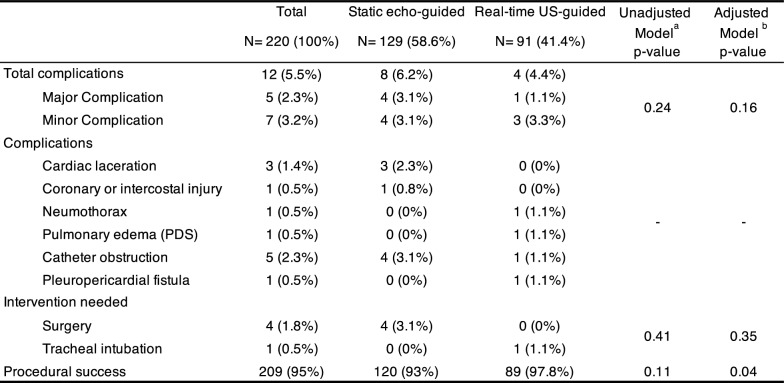
Outcomes

PDS Pericardiotomy Decompression Syndrome

^a^Univariate unadjusted analysis

^b^Poisson regression model adjusted by overlap weights (IRR 1.06 [95%CI 0.98–1.16], *P* = 0.163)

The results from the weighted Poisson regression demonstrated that the real-time US-guided technique was not associated with a statistically significant difference in the rate of total complications compared to the standard static echo-guided technique (incidence rate ratio [IRR] 1.06 [95% CI 0.98–1.16, p = 0.163]). Model diagnostics supported the appropriateness of the Poisson specification, with goodness-of-fit tests showing no evidence of overdispersion (deviance *χ*2 = 51.06, p = 1.000; Pearson χ2 = 31.19, p = 1.000). Expected values exhibited minimal variation (range: 16.74–17.78), with consistently small standard errors (0.008–0.043), indicating stable model predictions. The IRR for the need for intervention following complications was 0.35 (95% CI 0.04–3.13, p = 0.35), while the IRR for procedural success was 1.16 (95% CI 1.01–1.33, p = 0.04). Sensitivity analyses, including multiple imputation, worst-case scenario and complete case approaches, yielded consistent results (e-Table 3).

## Discussion

In this single-center retrospective study of 220 pericardiocentesis procedures, the overall complication rate was 5.5%. The real-time, in-plane US-guided approach had a complication rate of 4.4%, compared to 6.3% for the static echo-guided technique. Nevertheless, this difference was not statistically significant.

The overall complication rate observed in this cohort aligns with previously reported studies using echocardiography which range from 1.4 to 8% [1, 2, 15, 28–30]. Notably, among 91 real-time, in-plane US-guided pericardiocentesis, there was only one major complication involving a case of pulmonary edema that developed several hours after drainage and required less than 24 h of mechanical ventilation. Although rare, the occurrence of pulmonary edema after pericardiocentesis is documented in the literature, typically associated with pericardial decompression syndrome and sometimes related to rapid removal of large fluid volumes [24, 31–34]. In this patient, the initial fluid drainage was 400 cc, with a total volume of 720 cc drained over the first 24 h. It is possible that this complication was related more to the pathophysiology of decompression than to the technique itself.

In contrast, among 129 static echo-guided pericardiocentesis, there were four major complications (3.2%), including three cardiac lacerations and one thoracic vessel injury—all of which required emergency surgical interventions. Interestingly, the rate of major complications and failure rate with the static echo-guided technique in this study are slightly higher than previously reported (0.7–1.3% for major complications and 3–4%, for failure) [1, 3, 15, 29, 30]. While complication rates of up to 6% have been reported with the echo-guided approach, these were primarily associated with a subxiphoid approach [30].

The comparable overall complication rates between the two techniques in this cohort, and more importantly, the low rate of major complications paired with the high success rate with the real-time, in-plane US-guided approach, underscore the feasibility of this technique in emergency and critical care settings. Physicians in these environments are already trained in thoracic and cardiac ultrasound, and particularly in real-time ultrasound-guided central venous catheterization, where eye-hand-needle coordination skills are now routinely developed during residency and fellowship programs. This proficiency may facilitate timely and safe pericardial drainage for patients with critical cardiac tamponade. The procedural success rate was significantly higher with real-time ultrasound-guided pericardiocentesis. However, as this was not the primary outcome of our study, the interpretation of this secondary analysis remains limited.

To our knowledge, this study represents the largest cohort of real-time, in-plane, US-guided pericardiocentesis using a high frequency probe in an adult population. In 2021, Zhang et al. reported the use of this technique in 53 adult patients in a sitting position, achieving a 100% of success rate with no major complications [28]. The use of real-time US-guidance with different approaches, such as right parasternal or out-of-plane techniques, has also been described in recent case reports [35, 36]. It is noteworthy that most real-time, US-guided pericardiocentesis in our study were performed by physicians with limited experience in the technique, due to its relatively recent introduction and the cardiology staff’s previously limited exposure to real-time ultrasound-guided procedures using high-frequency linear probes and thoracic ultrasound. As with any procedure, an appropriate learning curve and extended experience are essential for minimizing complications and increasing success rates. Over time, critical care, emergency, and cardiology staff are likely to become increasingly comfortable with the technique, potentially leading to improved outcomes.

A further observation worth noting is that, not only the proportion of real-time ultrasound (US)-guided pericardiocentesis increased over time compared to the static technique, but the total number of procedures also increased. This trend can potentially be attributed to several factors, including improved procedure reporting through electronic medical records systems, but more importantly, the increase is likely driven by a growing number of critically ill patients admitted to our institution facilitated by the expansion of ICU bed availability over the past decade. Additionally, the enhanced perception of procedural safety and increased physician comfort with the real-time technique may have encouraged the drainage of smaller effusions with tamponade physiology that might have previously required open pericardial drainage.

Our study has some limitations. First, given the retrospective nature and the lack of randomization and blinding, our findings should be interpreted within the inherent limitations of the study design. Although regression with overlap weights-adjusted propensity scores was used to control for confounding, it was not possible to account for other non-identifiable biases and significant confounders that could influence complication risk or patient selection, such as specific echocardiographic findings including estimated pericardial effusion size, and the physician’s comfort level with real-time ultrasound guidance. Similarly, due to the retrospective nature of our study, some procedures may have been performed without proper documentation and thus not identified in our search. Additionally, complications may be underreported, potentially leading to an underestimation of their true incidence. A significant percentage of coagulation data was missing, which aligns with current clinical practice where routine coagulation tests are typically not required for most percutaneous procedures unless there is a specific concern about a patient’s coagulation status. To ensure the robustness of our findings, sensitivity analyses, including multiple imputation, worst-case scenario and complete case approaches, were performed and yielded consistent results (Supplementary Material file 1). Additionally, since coagulation abnormalities have not been associated with increased complication rates in previous studies, it is unlikely that the missing data had a significant impact on the study’s conclusions.

Our study suggests that real-time, in-plane, US-guided pericardiocentesis is a safe procedure. Nevertheless, it is important to note that our institution has an established ultrasound training program that adheres to current international training recommendations, [37–40] and proper training and skill development are fundamental to achieving good results. Consequently, these findings may not be replicable in other settings without similar training programs.

Finally, while our study suggests that there is no statistically significant difference in total complications between the real-time, US-guided and the static echo-guided pericardiocentesis techniques, the clinical significance in the difference of major complications might support the use of a real-time approach and highlights the need for future prospective research. Large-scale studies are, thus, required to fully evaluate the potential benefits of the real-time approach, particularly in terms of reducing major complications and improving overall success rates.

## Conclusions

In this single-center, retrospective cohort study, the real-time, in-plane US-guided pericardiocentesis technique was safe, with a total complication rate that was not significantly different from the static echo-guided approach. This finding highlights the potential applicability of this technique for timely and safe pericardiocentesis in emergent cardiac tamponade situations, particularly for critical care and emergency physicians who are already trained in real-time, in-plane ultrasound-guided procedures.

## Supplementary Information

Supplementary Material file 1.

Supplementary Material file 2: Video 1. Parasternal long axis. Anonymized.

Supplementary Material file 3: Video 2. In-plane Needle Insertion-Anonymized.

## Data Availability

The datasets used and analysed during the current study are available from the corresponding author on reasonable request.

## Abbreviations

CI: Confidence Interval
ICU: Intensive Care Unit
INR: International Normalized Ratio
IQR: Interquartile range
PS: Propensity Score
STROBE: Strengthening the Reporting of Observational studies in Epidemiology
US: Ultrasound
