# Supplementary material for: Complication rates in real-time ultrasound-guided vs static echocardiography-guided pericardiocentesis: a cohort study
Source: Echo Res Pract. 2025 Apr 1;12:8. doi: 10.1186/s44156-025-00071-6 (PMC11959931; doi:10.1186/s44156-025-00071-6)
Supplement: Supplementary file 1 — Supplementary Material file 1. [file 44156_2025_71_MOESM1_ESM.docx]

**Complication Rates in Real-time Ultrasound-guided vs Static Echocardiography-guided Pericardiocentesis: A Cohort Study**

**ELECTRONIC SUPPLEMENTARY MATERIAL**

**(ESM)**

Virginia Zarama, MD., MSc.^1,2^, Carlos E. Vesga, MD.^2,3^, John Balanta-Silva, MD ^2^, Mario M. Barbosa, MD., MSc.^4^, Jaime A. Quintero, MD.^4^, Ana Clarete MD.^4^, Paula A. Vesga-Reyes, MD.^2^, Juan Carlos Silva Godinez, MD., MPH., MSc.^5,6^

1. Departamento de Medicina Crítica, Fundación Valle del Lili, Cali, Colombia.
2. Facultad de Ciencias de la Salud, Universidad Icesi, Cali, Colombia
3. Departamento de Cardiología, Fundación Valle del Lili, Cali, Colombia
4. Centro de Investigaciones Clínicas (CIC), Fundación Valle del Lili, Cali, Colombia
5. Harvard T.H. Chan School of Public Health, ECPE Department, PPCR Program, Boston, United States
6. Escuela Nacional Colegio de Ciencias y Humanidades, Universidad Nacional Autónoma de México, Ciudad de México, México.

Corresponding Author: Virginia Zarama, MD., MSc.

Department of Emergency Medicine

Fundación Valle del Lili - Universidad Icesi

Carrera 98 # 18-49, Cali 760032, Colombia

Email: virginia.zarama@fvl.org.co

Tel (+57) 2 -331-9090 – ext.3277

TABLE OF CONTENTS

[1. STRENGTHENING THE REPORTING OF OBSERVATIONAL STUDIES IN EPIDEMIOLOGY 3](#_Toc175816203)

[1.1. e-Table 1. STROBE Checklist 3](#_Toc175816204)

[2. ULTRASOUND TRAINING INFORMATION 4](#_Toc175816205)

[3. OVERLAP WEIGHTS 4](#_Toc175816206)

[3.1. e-Figure 1. Density of propensity scores by treatment status and after overlap weights adjusted distribution 4](#_Toc175816207)

[4. MISSING DATA 5](#_Toc175816208)

[4.1. e-Figure 2. Plot: Missing data for each variable 5](#_Toc175816209)

[4.2. Missingness Mechanism 5](#_Toc175816210)

[4.3. Multiple imputation modeling 6](#_Toc175816211)

[5. SENSITIVITY ANALYSES 6](#_Toc175816212)

[5.1. e-Table 2. Sensitivity Analyses 6](#_Toc175816213)

[6. REFERENCES 7](#_Toc175816214)

## STRENGTHENING THE REPORTING OF OBSERVATIONAL STUDIES IN EPIDEMIOLOGY

### e-Table 1. STROBE Checklist

## ULTRASOUND TRAINING INFORMATION

Ultrasound training program in our institution consists of 40 hours of theoretical learning including standard lectures and literature review for at-home review, either traditionally or guided by virtual learning environment platform. Practical learning is performed initially in simulators and healthy models and afterwards competency on cardiac, lung, abdominal and vascular ultrasound is achieved during approximately 160 hours of supervised hands-on practice during a one-month rotation (residents or fellows) or 3-month part-time diplomate (attendings). At the end of training, a minimum of 10 supervised real-time, in-plane, US-guided vascular access procedures are completed, but usually around 25-30 procedures are performed.

## OVERLAP WEIGHTS

### e-Figure 1. Density of propensity scores by treatment status and after overlap weights adjusted distribution


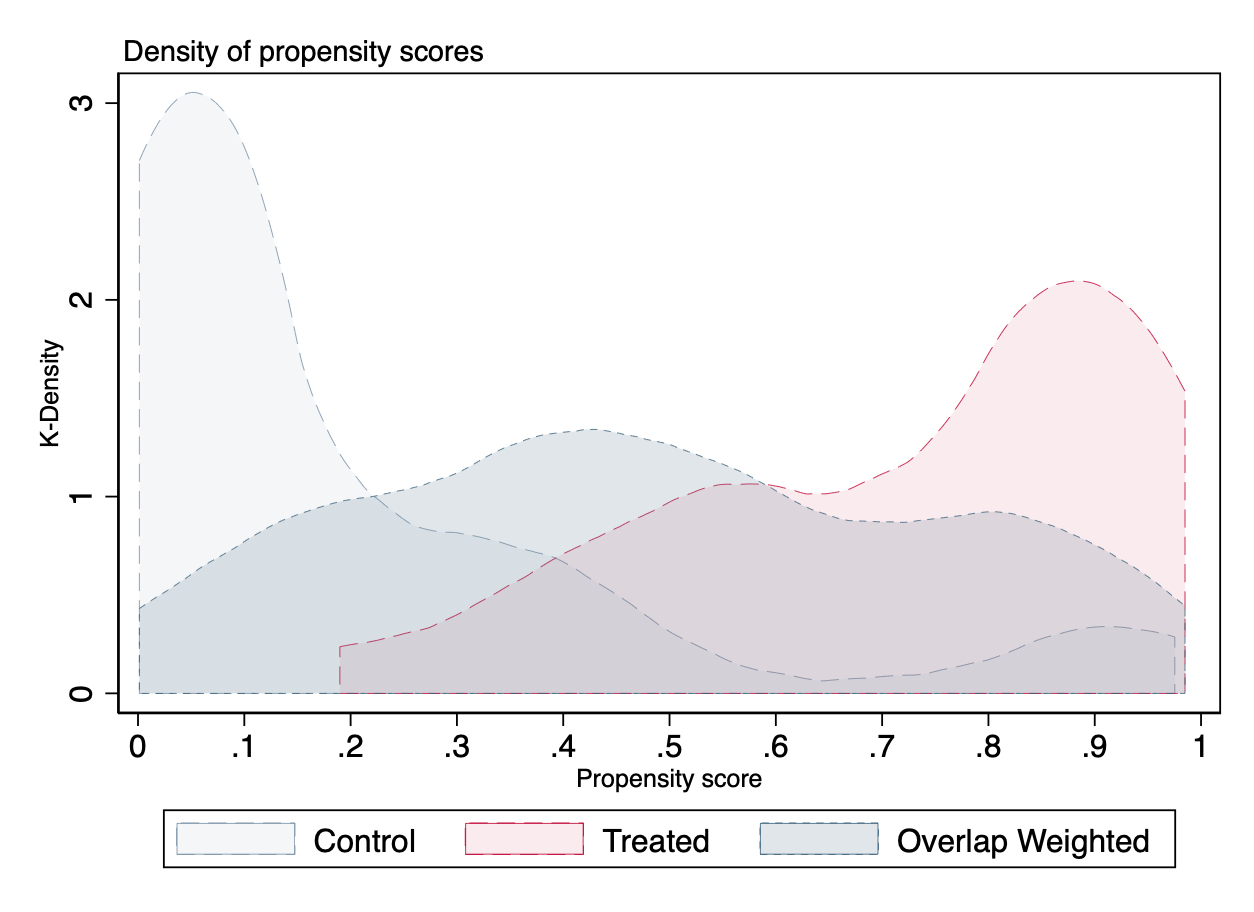


## MISSING DATA

### e-Figure 2. Plot: Missing data for each variable


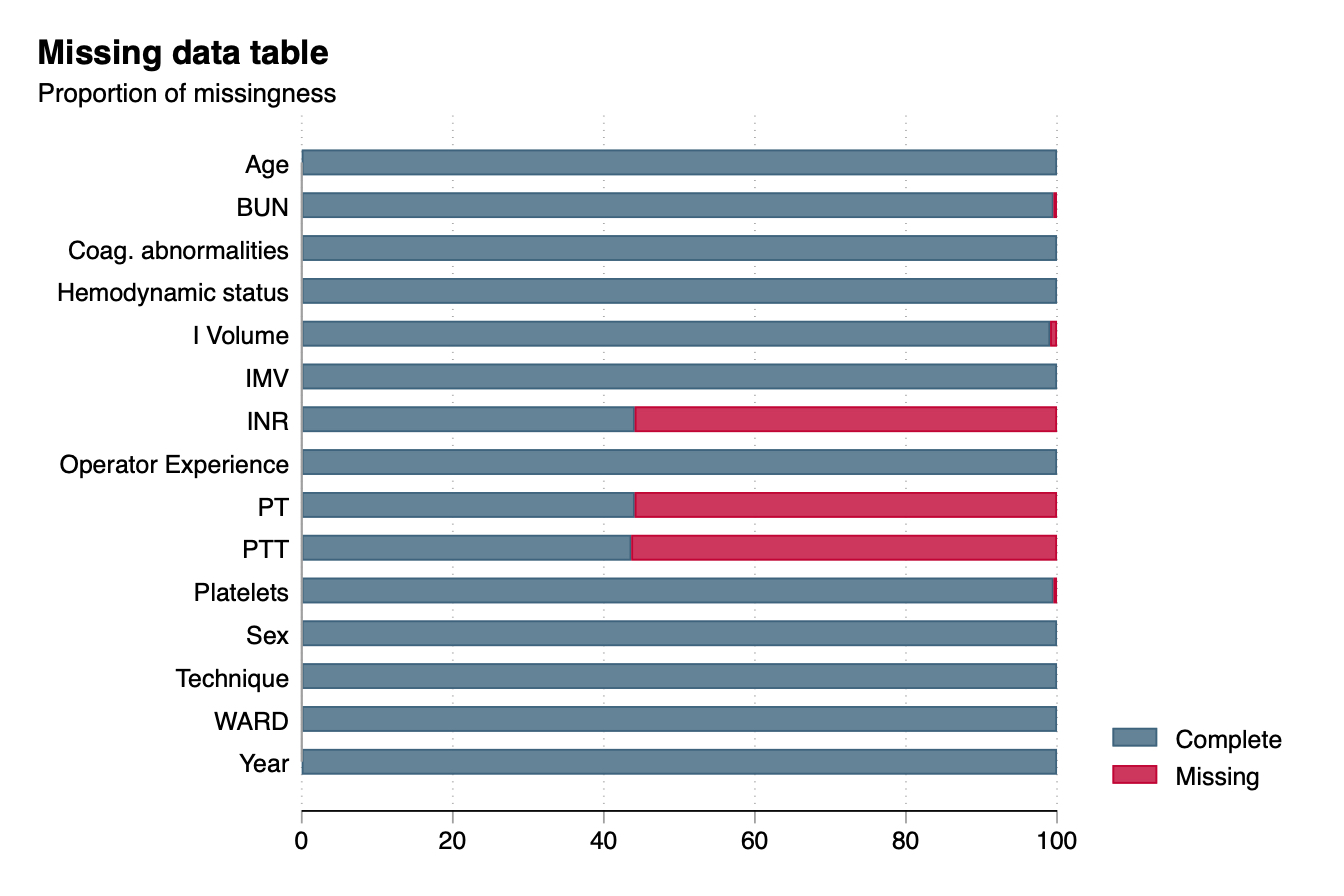


### Missingness Mechanism

Missing data primarily pertained coagulation tests, which aligns with current clinical practice, as routine coagulation testing is generally not required for percutaneous procedures. An analysis of the distribution of missing variables showed that the missingness was independent of the outcome but was not completely random (MCAR). Instead, the missing data were associated with certain observed variables—primarily the procedure year, hospital ward, and technique used—suggesting that a missing at random (MAR) mechanism is more appropriate. To evaluate the MAR assumption, Little's test was conducted, which rejected the null hypothesis further supporting the MAR mechanism (p = 0.001),^1^ ).^2,3^ . Since observed data alone cannot distinguish between MAR and MNAR, sensitivity analyses were conducted as recommended to assess the potential impact of MNAR on the estimated results.^4,5^

### Multiple imputation modeling

Missing data for pT, pTT, INR, BUN, platelet count and initial volume drainge were imputed using multiple imputation by chained equations (MICE) in Stata/BE 18.5 (StataCorp, TX: StataCorp LLC). MICE was chosen because multiple variables had missing values, the data did not follow normal distributions, and the missingness was non-monotonic.^6^ Outcome data was available for all patients. Coagulation tests were available for 44% of the observations, platelet count and blood nitrogen urea were missing in one observation, and initial drainage volume was missing in 2 procedures. All missing variables showed a non-normal distribution, therefore, imputation modeling was conducted with predictive mean matching (pmm).^7-9^ Overall, 56% of patients had some missing data.^10^ The imputation procedure started with 50 imputations and after applying the quadratic rule described by VanHipple, another 60 imputations were added,^11^ knn (k-nearest neighbor) = 5. Variables included in the multiple imputation model were: the outcome variable^12^, all variables used in the primary analysis, and all possible variables that may predict missingness, (this reduces the chance of inadvertently omitting an important cause), ^4,13^ as recommended. ^4,14-16^ The latter were chosen based on clinical background and statistically driven from the comparison of missing data vs non-missing data difference. Variables included were age, sex, etiology, hospital ward, invasive mechanical ventilation, hemodynamic instability, cardiac tamponade, technique, year of the procedure, operator experience and operator. Descriptive statistics, histograms and kernel-density estimation graphics were used to compare the imputed and observed data, finding appropriate distributions of imputated values.^17^

## SENSITIVITY ANALYSES

### e-Table 2. Sensitivity Analyses

## REFERENCES

1. Bhaskaran K, Smeeth L. What is the difference between missing completely at random and missing at random? *International Journal of Epidemiology*. 2014;43(4):1336-1339. doi:10.1093/ije/dyu080

2. Little RJA. A Test of Missing Completely at Random for Multivariate Data with Missing Values. *Journal of the American Statistical Association*. 1988/12/01 1988;83(404):1198-1202. doi:10.1080/01621459.1988.10478722

3. Li C. Little's Test of Missing Completely at Random. *The Stata Journal*. 2013/12/01 2013;13(4):795-809. doi:10.1177/1536867X1301300407

4. Sterne JA, White IR, Carlin JB, et al. Multiple imputation for missing data in epidemiological and clinical research: potential and pitfalls. *BMJ*. Jun 29 2009;338:b2393. doi:10.1136/bmj.b2393

5. Jakobsen JC, Gluud C, Wetterslev J, Winkel P. When and how should multiple imputation be used for handling missing data in randomised clinical trials - a practical guide with flowcharts. *BMC Med Res Methodol*. Dec 6 2017;17(1):162. doi:10.1186/s12874-017-0442-1

6. !!! INVALID CITATION !!! ￼;

7. Marshall A, Altman DG, Royston P, Holder RL. Comparison of techniques for handling missing covariate data within prognostic modelling studies: a simulation study. *BMC Med Res Methodol*. Jan 19 2010;10:7. doi:10.1186/1471-2288-10-7

8. Cro S, Morris TP, Kenward MG, Carpenter JR. Sensitivity analysis for clinical trials with missing continuous outcome data using controlled multiple imputation: A practical guide. *Stat Med*. Sep 20 2020;39(21):2815-2842. doi:10.1002/sim.8569

9. Lee KJ, Carlin JB. Multiple imputation in the presence of non-normal data. *Stat Med*. Feb 20 2017;36(4):606-617. doi:10.1002/sim.7173

10. Madley-Dowd P, Hughes R, Tilling K, Heron J. The proportion of missing data should not be used to guide decisions on multiple imputation. *J Clin Epidemiol*. Jun 2019;110:63-73. doi:10.1016/j.jclinepi.2019.02.016

11. von Hippel PT. How Many Imputations Do You Need? A Two-stage Calculation Using a Quadratic Rule. *Sociological Methods & Research*. 2020/08/01 2018;49(3):699-718. doi:10.1177/0049124117747303

12. Huque MH, Carlin JB, Simpson JA, Lee KJ. A comparison of multiple imputation methods for missing data in longitudinal studies. *BMC Medical Research Methodology*. 2018/12/12 2018;18(1):168. doi:10.1186/s12874-018-0615-6

13. Collins LM, Schafer JL, Kam CM. A comparison of inclusive and restrictive strategies in modern missing data procedures. *Psychol Methods*. Dec 2001;6(4):330-51.

14. Zhang Z. Multiple imputation with multivariate imputation by chained equation (MICE) package. *Ann Transl Med*. Jan 2016;4(2):30. doi:10.3978/j.issn.2305-5839.2015.12.63

15. White IR, Royston P, Wood AM. Multiple imputation using chained equations: Issues and guidance for practice. *Stat Med*. Feb 20 2011;30(4):377-99. doi:10.1002/sim.4067

16. Moons KG, Donders RA, Stijnen T, Harrell FE, Jr. Using the outcome for imputation of missing predictor values was preferred. *J Clin Epidemiol*. Oct 2006;59(10):1092-101. doi:10.1016/j.jclinepi.2006.01.009

17. Nguyen CD, Carlin JB, Lee KJ. Model checking in multiple imputation: an overview and case study. *Emerg Themes Epidemiol*. 2017;14:8. doi:10.1186/s12982-017-0062-6
